# Palliative Electrochemotherapy in Vulvar Carcinoma: Preliminary Results of the ELECHTRA (Electrochemotherapy Vulvar Cancer) Multicenter Study

**DOI:** 10.3390/cancers11050657

**Published:** 2019-05-12

**Authors:** Anna Myriam Perrone, Andrea Galuppi, Cecilia Pirovano, Giulia Borghese, Piero Covarelli, Francesca De Terlizzi, Martina Ferioli, Silvia Cara, Alessio Giuseppe Morganti, Pierandrea De Iaco

**Affiliations:** 1 Oncologic Gynaecology Unit, Department Medical and Surgical Sciences (DIMEC), S. Orsola-Malpighi Hospital, University of Bologna, Via Massarenti 13, 40138 Bologna, Italy; giuliamaria.borghese@gmail.com (G.B.); craslv@gmail.com (S.C.); pierandrea.deiaco@unibo.it (P.D.I.); 2 Radiation Oncology Centre, Department of Experimental, Diagnostic and Specialty Medicine, University of Bologna, Via Massarenti 13, 40138 Bologna, Italy; andrea.galuppi@aosp.bo.it (A.G.); m.ferioli88@gmail.com (M.F.); amorganti60@gmail.com (A.G.M.); 3 Department of Obstetrics and Gynaecology, ASST Lecco Ospedale Manzoni, Via dell’Eremo 9/11, 23900 Lecco, Italy; c.pirovano@asst-lecco.it; 4 General and Oncologic Surgery Unit, Department of Surgical and Biomedical sciences, Ospedale Santa Maria della Misericordia, University of Perugia, Piazza Menghini, 1, 06129 Perugia, Italy; piero.covarelli@unipg.it; 5 Scientific & Medical Department IGEA S.p.A. Via Parmenide 10/A, 41012 Carpi (Mo), Italy; f.deterlizzi@igeamedical.com

**Keywords:** vulvar cancer, recurrence, electrochemotherapy, bleomycin, palliative therapy

## Abstract

Vulvar cancer (VC) is a rare disease of which recurrence poses management problems due to patients’ advanced age and comorbidities, and to the localization of the disease. Palliative treatments, allowing local disease control in patients previously treated with multimodal therapies or with comorbidities, are lacking. In this study we tested electrochemotherapy (ECT) on recurrent VC refractory to standard therapies to assess the tumor response and to define the selection criteria for patient’s candidate to ECT. This is a multicenter observational study carried out in five Italian centers. Data about patients and tumor characteristics, treatment, toxicity, and clinical response were recorded. In all procedures, intravenous bleomycin was administered according to European Standard Operative Procedure ECT (ESOPE) guidelines. Sixty-one patients, with a median age 79 years (range: 39–85) and mainly affected by squamous cellular carcinoma (91.8%), were treated with ECT. No serious adverse events were reported. Patients were discharged after three days (median, range: 0–8 days). Two months after ECT, the clinical response rate was 83.6% and was not related to age, body mass index, International Federation of Gynecology and Obstetrics (FIGO) stage, number of treated nodules, or previous treatments. ECT is a safe procedure with a favorable cost-effectiveness ratio and should be considered as a treatment option for local disease control in patients unsuitable for standard therapies.

## 1. Introduction

Vulvar cancer (VC) is a rare disease accounting for 5% of all gynecological malignancies, with a higher incidence in older women. However, during recent decades, the incidence of VC has increased in young women due to the spread of Human Papilloma Virus (HPV) infection. Squamous cellular carcinoma (SCC) represents the most common histotype (90% of cases), even if other less-frequent histological types (melanoma, extra-mammary Paget disease, Bartholin gland adenocarcinoma, verrucous carcinoma, basal cell carcinoma, and sarcoma) are reported [1]. Patients with VC are treated with neoadjuvant or first-line chemo-radiation, surgery, or other multimodality treatments according to stage, site of disease, and performance status [2]. Despite this, the disease recurs in about 33% of cases with around 70% having a five-year survival rate [3]. In case of recurrence, therapeutic options are limited and quality of life is poor. Therefore, the development of new therapies is needed [4].

Electrochemotherapy (ECT) represents a new therapeutic option that, during the last few years, has expanded to different clinical settings. In fact, the procedure was initially introduced to treat small metastatic cutaneous tumors not amenable to surgery or radiation therapy [5]. However, the codification of the procedure (European Standard Operative Procedure ECT, ESOPE 2006) [6] as a safe application in clinical practice, the progressive clinical experience, and technological improvements have extended the use of ECT to new groups of patients. As an example, the National Institute for Health and Care Excellence (NICE) has recognized ECT as an effective treatment option for melanoma skin metastases, and skin basal and squamous cell carcinoma [7,8]. Reversible electroporation, induced by the short electric pulses, increases cancer cell membranes’ permeability to low-permeant cytotoxic agents like bleomycin (BLM) and cisplatin (CDDP), strengthening their therapeutic effect. The procedure has gradually become widespread in many European centers (about 150). Several studies have confirmed ECT efficacy in superficial skin tumors and have suggested a possible role in the treatment of mucosal and deep-seated tumors of various histological types [9,10,11,12,13].

In 2013, we published the first report on the use of palliative ECT on nine patients with VC who relapsed after multimodality treatments and were unsuitable for standard therapies. The results were encouraging, with a 62.5% complete response (CR) rate and relevant symptoms relief (pain, bleeding, bad smell, and urinary discomfort) [14]. ECT was further tested in 25 other patients, obtaining similar promising results. VC local control was achieved in about 80% of cases, with a 48% CR rate, and 7 out of 25 patients underwent a second course of ECT for disease progression, achieving a 43% CR rate [15]. Similar results from a series of 13 patients were reported by another Italian Group [16]. Based on these results, several Italian centers started a scientific cooperation on this setting of VC patients. Data from the different centers were collected in a National Database named ELECTRA (Electrochemotherapy vulvar cancer), coordinated by the Gynaecologic Oncology Unit of Bologna University.

The aim of this multicenter observational study is to report on the oncological outcome as a local control of the disease, achieved with the use of ECT in the treatment of VC refractory to standard treatment in a larger patients’ series. The secondary endpoint is to identify prognostic factors in order to improve patients’ selection for ECT.

## 2. Materials and Methods

### 2.1. Study Design, Inclusion Criteria and Data Collection

This observational study was partly retrospective and partly prospective. The study was approved by the Ethical Committee of Bologna (EC code 42/2013/O/OssN), and patients signed the consensus to data analysis. Women with relapsed VC treated with ECT were included in the analysis. Patients’ data was collected using a web-based electronic Case Report Form (e-CRF). The collected information was anonymized, encrypted, and finally sent to the remote server for data storage. In this way, data about patients and their treatment characteristics were registered in a standardized and consistent way among different centers and physicians. In particular, the following data were recorded: Age, BMI, comorbidities, histologic type, stage, previous medical, surgical, and radiotherapy treatments and number, site, and size of recurrences. If available, a photographic image of the lesions before and after ECT was collected and uploaded. From our clinical records, we selected a cohort of patients with VC relapse treated with multimodal therapies as a control group. These women were submitted to palliative therapies before the introduction of ECT in our department.

### 2.2. ECT Procedure, Response Evaluation and Follow Up

ECT was performed according to ESOPE guidelines [6]. Electric pulses were delivered using a pulse generator certified for clinical application (Cliniporator, IGEA, Carpi, Italy). All the information about the type of chemotherapeutic agent, electrode used, as well as details about the area covered by the electric field during each procedure and operation times were recorded in the database. Clinical response to ECT was evaluated based on the (Response Evaluation Criteria in Solid Tumors (RECIST) [17] criteria 40–60 days after the procedure. Complete response (CR) was defined as the disappearance of the target lesion, and partial response (PR) as a decrease of at least 30% of its maximum diameter. Progressive disease (PD) was defined as an increase of at least 20% of the maximum diameter. All responses defined by neither CR–PR nor PD criteria were categorized as a stable disease (SD). The RECIST criteria were applied to all visible lesions. Data about follow-up were collected, and the patients were clinically re-evaluated every six months, or in cases of symptoms recurrence/progression. All adverse events were recorded.

### 2.3. Statistical Analysis

Statistical analysis was performed using IBM SPSS Statistics 25.0 software (IBM Corp., Armonk, NY, USA). Continuous variables are presented as medians and ranges. Categorical data are expressed as percentages and absolute rates. Statistical comparisons of response rates were performed using contingency tables and the Pearson chi square test. Survival curves were calculated using the Kaplan–Meier method. The log-rank test was used to compare survival curves. Local progression-free survival (LPFS) was calculated from the date of treatment to the one of relapse/progression in the treated area, or to the last follow-up.

## 3. Results

### 3.1. Patients and Tumor Characteristics

**Figure 1:**
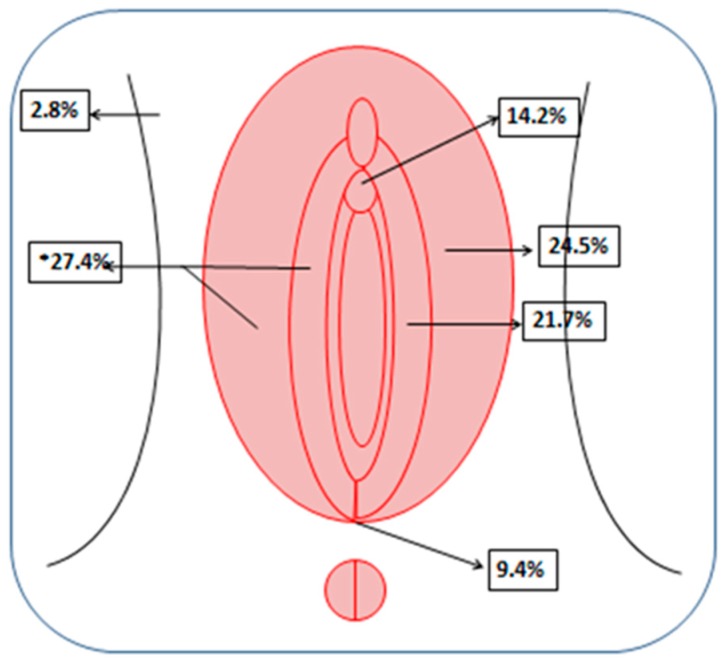
Topographic distribution of the treated lesions.

Sixty-one patients affected by VC refractory to standard therapies were treated with ECT and included in the analysis (Bologna: 47, Lecco: 7, Perugia: 4, Naples: 2, and Bergamo: 1). The patients’ median age and Body Mass Index (BMI) were 79 years (range: 39–85) and 25 (range: 15–39), respectively. Their tumors’ histological types included SCC (57 patients, 91.8%), Paget’s disease (three patients, 6.6%), and malignant melanoma (one patient, 1.6%). Considering all the treated patients, 37 presented single nodules (60.7%) and 24 (39.3%) presented multiple nodules. In total, 106 nodules were treated (range of number of nodules per patient: 1–7). The median diameter of the lesions treated was 20 mm (range: 1–110 mm). The topographic distribution of the nodules treated is shown in Figure 1. The lesions were equally distributed between the left (46.2%) and right sites (53.8%).

### 3.2. Procedure and Toxicity

Forty-eight (79%) patients underwent ECT under general anesthesia, while 13 (21%) patients were treated under local anesthesia. All procedures were performed following ESOPE guidelines, and we were able to cover the whole area of the lesions in 105 treated nodules (99.1%). Intravenous bleomycin was administered in all cases. The linear electrode was used in 94 lesions (88.7%), the finger electrode in seven lesions (6.6%), whereas the hexagonal electrode was chosen for five lesions (4.7%). No other concomitant treatments were performed. No treatment-related serious adverse events were reported. Short-term toxicity was evaluated immediately after procedure and at the first follow-up; long-term toxicity was then evaluated at the subsequent follow-up. The most common local side effect was erythema (20%), hyperpigmentation (6%), skin ulceration (1%), and mild pain (24%) which was easily controlled by the administration of minor analgesics. Patients were discharged from the hospital after a median time of three days (range: 0–8 days).

### 3.3. Tumor Response

**Figure 2:**
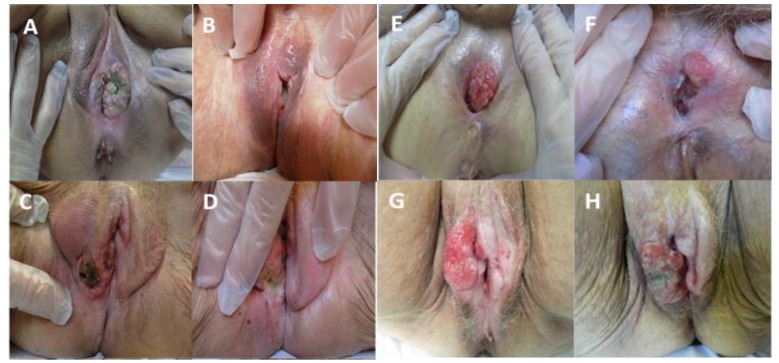
(**A**,**C**,**E**,**G**) nodules before Electrochemotherapy treatment; (**B**,**D**,**F**,**H**) nodules two months after ECT; (**B**) complete clinical response; (**D**) partial clinical response; (**F**) stable disease; and (**H**) progressive disease.

Response to therapy was assessed two months after ECT. Tumor response was evaluated for only 55 patients, because six patients did not reach the first follow-up evaluation; 4 of 6 patients died because of systemic disease progression and 2 of 6 were lost to follow-up. In all evaluable patients, the clinical overall response (CR+PR) rate was 83.6%. CR was recorded in 29 cases (52.7%), PR in 17 (30.9%), SD in 6 (10.9%), and PD in 3 (5.5%) (Figure 2). Considering the response per nodule (total number of nodules evaluated: 96), the objective response rate was 86.9%. By analyzing our results, adopting an intent-to-treat analysis, and considering as non-responders the six patients lost at follow-up, we report clinical overall response (CR+PR) in 46 patients (75.4%), and no clinical response (SD+PD) in 15 patients (24.6%). The overall clinical response (CR+PR) rate for the SCC subgroup was 82%.

The correlation between response to ECT per patient and different factors such as age, BMI, FIGO stage, and number of nodules was analyzed. Similarly, the correlation between response per nodule with size, previous treatments, and site localization was analyzed. None of the following parameters influenced the response to treatment: age; BMI; FIGO stage; number, site and dimension of nodules; and previous treatments (Table 1).

### 3.4. Patient Outcome

**Figure 3:**
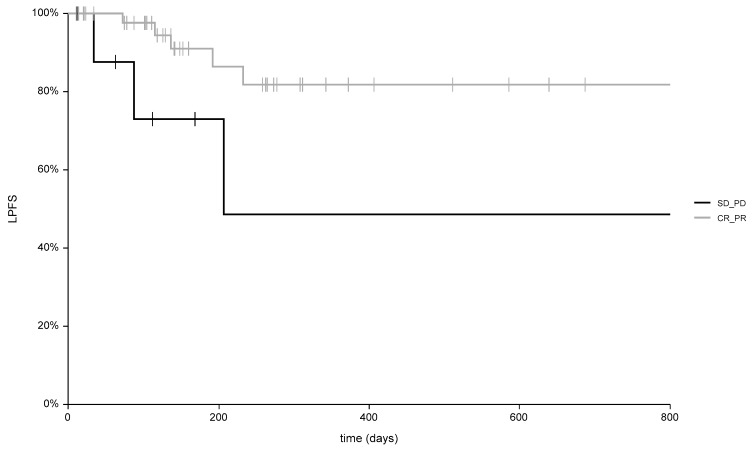
Different local control of disease (*p* = 0.007) in patients with complete response (CR) and partial response (PR), compared to patients with stable disease (SD) and progressive disease (PD). (LPFS = local progression-free survival).

Considering all patients, the median follow-up was 142 days (range: 40–1524 days). Among the 29 complete responder patients, the median follow-up was 258 days (range: 40–1524 days). Six patients died of disease after a median time of 334 days (range: 78–586 days) and two patients experienced disease recurrence 136 and 192 days after ECT. Patients with disease recurrence were referred to palliative care. For the 17 partial response patients, the median follow up was 126 days (range: 40–1278 days). In this subgroup, five patients died after a median time of 101 days for systemic progression (range: 40–141 days), two patients were referred to other treatments (Imiquimod and nodulectomy), and five patients underwent a second ECT course with an additional response to treatment, including two patients with CR, two patients with PR, and one patient who died from systemic disease progression. Among the six patients with no change in tumor size (SD), the median follow-up was 88 days (range: 40–1440 days), with two patients who died after 168 and 192 days, respectively, for systemic disease progression. In Figure 3, the local progression-free survival curves are reported for objective response patients (CR+PR) and non-responding patients (SD+PD).

**Figure 4:**
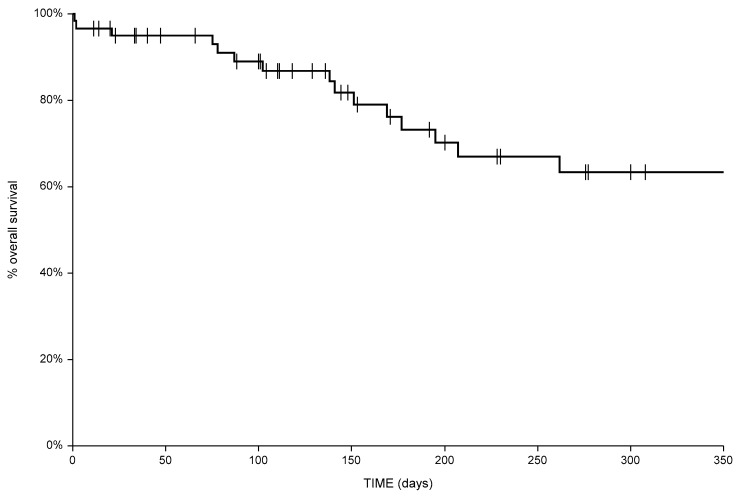
Overall survival for the total cohort of patients.

The six-month local progression-free survival was 91% (C.I. 81–100%) and 73% (C.I. 41–100%) in responders and non-responding patients, respectively (*p*: 0.007). Overall survival for the entire cohort of patients is shown in Figure 4. The six-month overall survival was 72%.

The analysis of our clinical records found nine patients with clinical features similar to our study population. The median age was 82 years (range: 57–87 years). Previous treatments were surgery in all patients, combined with radiotherapy (seven patients) or chemoradiation (two patients). Upon relapse, all patients were treated with chemotherapy (weekly paclitaxel). In four patients, the treatment was prematurely stopped due to toxicity and, in three patients, due to early progressive disease. Overall, 0/9 (0%) patients showed CR, 2/9 (22%) had PR, 4/9 (45%) showed SD, and 3/9 (33%) had PD. We did not perform actuarial analysis of local control and overall survival due to the small sample size.

## 4. Discussion

Our group was the first to test the local efficacy of ECT in refractory cases of VC, and here we present the largest study cohort on the use of palliative ECT in this group of patients. In this study, we analyzed only the outcome of the response to therapy. The present results add new evidence to previous reports [14,15,16], showing: (i) ECT is a safe procedure even in centers without a long experience (as evidenced by the absence of severe toxicities also in centers where only a small number of patients were treated); and (ii) ECT has a favorable cost-effectiveness ratio due to the low cost of BLM and the short period of hospitalization. For these reasons, ECT should be considered as a treatment option for these patients.

We introduced ECT in the management of refractory VC for several reasons, mainly because of the many challenges related to the treatment of these neoplasms. These challenges include VC site (near to urethra, rectum, and vagina), multifocality, and high incidence in elderly patients. An oncological radical resection can cause disfiguring consequences, while concurrent chemo-radiotherapy can produce significant acute toxicity and potential late complications [2]. Tumor-related pain, burning, bleeding, pruritus, dysuria, and odor can significantly affect the quality of life of the patients, and effective supportive therapies which are applicable as medications capable of significantly relieving these symptoms are lacking [14].

In our experience, the procedure was at first tested in older patients and then progressively offered to all patients requiring a palliative treatment. Our study confirmed the efficacy of ECT, strengthening the results of previous studies. Importantly, in the management of skin cancers, ECT allows the local control of the disease of VC and, if needed, can be administered concomitant to, or following, other treatments such as radiotherapy or systemic therapies in patients with distant metastases.

Identification of patient- and tumor-related predictive factors for responses to ECT should be important in selecting the best candidates for this procedure. Therefore, all clinical-related aspects that could influence ECT activity were studied.

Surprisingly, no statistical difference in terms of response to treatment in relation to histology, site, dimensions, number of nodules, and previous treatments was found. Moreover, no difference was observed between patients with SCC (57) versus patients with other types of cancer histology (three Paget disease and one malignant melanoma). These results, within the limitation of some small subgroups in patients’ analyses, seems to confirm previous observations of a tumor response not related to tumor histology, as reported in literature for other tumors. In fact, several analyses reported that ECT is equally effective in basal cell carcinoma, malignant melanoma, skin recurrences from breast cancer, squamous cell carcinoma, Kaposi’s sarcoma, Merkel cell carcinoma, and other tumors [11].

Furthermore, no correlation was found between the response to treatment and the tumor’s site. The possible explanation is that even though some areas are more difficult to treat than others for anatomical reasons when ECT was performed according ESOPE guidelines and all the tumor’s area was covered by the treatment, the tumor response could be the same regardless of distance from urethra, vagina, or anus.

In literature, controversial data about the correlation between the response to ECT and the size and number of skin cancer nodules treated is reported. Some studies showed no statistical difference among the responses when tumors were grouped according to their size [18,19], and others studies reported that a tumor size equal to or less than 3 cm, and a number of lesions equal to or less than 20, were associated with a better response to treatment [20,21]. Regarding the number of nodules treated, some authors support the hypothesis that the non-responsive nodules were electroporated after the 28 min recommended by the guidelines [22,23], although the latest studies would show a BLM effect even up to 40 min [10]. In our analysis, no statistical difference was observed among the responses when tumors were divided according size and number of nodules, but the maximum number treated in a session of ECT was seven. The small number of lesions guarantees the possibility that all the lesions have been electroporated beyond the recommended BLM washout time.

No correlation was observed between responses and previous treatments; this means that the histological modification of tissues due to radiotherapy does not compromise the efficacy of ECT treatment. Probably, the real mechanism underlying the ECT response is little understood. High drug concentrations led to cellular toxicity and death. ECT combines the ability to exert a cytotoxic effect on cancer cells with the capability to generate an anticancer immune response. In fact, some studies demonstrated that the local effect of ECT is reduced in immunodeficient mice with a lack of T lymphocytes, and interleukin-2 or other pro-inflammatory cytokines [24]. This immune-mediated effect could explain the lack of response in some patients (SD 10.9–PD 5.5%) and could make the treatment unsuitable for immune-compromised patients. No patients with immune-suppressive diseases such HIV or similar were present in our records, but this issue should be more extensively studied in the future, particularly in non-responders. The question about the cause of the failure of treatment remains open.

BLM and cisplatin (CDDP) are the most-used drugs for ECT [9]. Based on the wide experience reported and available in literature when we started the treatments, we chose to employ intravenous BLM. BLM is a water-soluble antibiotic causing toxicity to mammalian cells by means of single- and double-stranded breaks in DNA that ultimately lead to cell death. Electro-permeabilization of cells allows the entry of the drug in the cytosol causing the damage despite low blood concentration [11,25,26]. ECT with CDDP has achieved efficacy for palliative treatment of malignant melanoma [27,28], squamous cell carcinoma [29], basal cell carcinoma [29], and cutaneous lesions of breast cancer [30]. Moreover, the response to therapy with CDDP was proven to be dependent on the drug dose, the amplitude of the electric pulses, and the sequencing and time interval of CDDP administration [31,32]. Therefore, CDDP seems less easy to manage than BLM, especially in unfit patients such as those proposed for palliative care. The possibility to test CDDP in patients such as these, as an alternative to BLM, presents an attractive issue for future studies.

It is very difficult to compare our group of patients treated with ECT with others submitted to palliative treatments in our Institution, because before the introduction of ECT most patients in this setting received only supportive therapy. Therefore, we were able to find only a very small group of patients treated with chemotherapy. Despite the small sample size of our control group, the superiority of ECT in terms of clinical response seems evident. Obviously, a more reliable comparison would require the design and execution of a prospective comparative trial.

## 5. Conclusions

Based on these results, and considering the poor alternatives available for the management of VC, we suggest ECT as a new therapeutic tool for patients unsuitable for surgery, for chemo-radiotherapy-resistant lesions, and for patients with severe comorbidities and/or of advanced age who have already received the available standard treatments. Therefore, Oncological Societies should consider this therapy as one of the options in palliative care for vulvar cancer.

The procedure is easy and quick to perform (25–30 min) and is associated with a short hospital stay. For these reasons, and due to the low price of the drugs used, ECT has a favorable cost-effectiveness ratio. The side effects are minor, and most patients require only low dose of analgesics to treat ECT-related pain. Moreover, unlike radiotherapy, it is possible to repeat several ECT cycles without precluding other types of treatment.

Further studies are required to (i) evaluate the risk of pulmonary complications produced by BLM in the case of multiple ECT, (ii) analyze the possibility of severe ulcers mainly after the treatment of large tumors, and (iii) identify the best candidates for this promising treatment.

Finally, based on the significant correlation between clinical response and local control, further trials investigating the possibility of improving the response rate are justified. These studies could be based on the combination of ECT with other therapies.

## Figures and Tables

**Table 1:** Correlation between response to ECT according to different covariates: age; BMI; stage of disease; number, site and dimension of nodules; and previous treatments. CR = complete response; PR = partial response; SD = stable disease; PD = progression disease; RT = radiotherapy; CHT = chemotherapy.

Patient’s Characteristics	CR+PR		SD+PD		*p*
	*n*	%	*n*	%	
Age					
<80	26	86.7	4	13.3	0.716
≥80	20	80.0	5	20.0	
BMI					
<25	25	86.2	4	13.8	0.170
≥25	21	80.8	5	19.2	
Stage					
FIGO I	22	92.0	2	8.0	
FIGO II	2	66.7	1	33.3	0.442
FIGO III	6	86.0	1	14.0	
FIGO IV	1	50.0	1	50.0	
Unknown	15	78.9	4	21.1	
Number of nodules					
=1	28	93.4	2	6.6	0.063
>1	18	72.0	7	28.0	
Site					
Vulva	62	88.6	8	11.4	
Paraurethral region	14	100.0	0	0	0.167
Posterior commisture	9	90.0	1	10.0	
Inguinal region	1	50.0	1	50.0	
Dimension of nodules (cm)					
≤3	64	90.1	7	9.9	0.702
>3	20	87.0	3	13.0	
Previous treatments					
RT	1	50.0	1	50.0	
Surgery (single or multiple)	25	83.3	5	16.7	
Surgery + RT	4	80.0	1	20.0	0.762
Surgery + CHT	1	100.0	0	0.0	
RT + CHT	5	100.0	0	0.0	
Surgery + RT + CHT	2	100.0	0	0.0	
Palliative/no treatment	8	80.0	2	20.0	
